# Gel-Based Drug Delivery Platforms: A Critical, Mechanistic Review of Design, Cross-Linking, and Disease-Specific Translation (2010–2026)

**DOI:** 10.3390/gels12090787

**Published:** 2026-09-01

**Authors:** Rama Rao Nadendla, Venkata Suresh Ponnuru, Pallavi Vadlamudi, Koora Narasimhulu Rajini Kanth, Mohan Chandu Uppalapati, Koushik Yetukuri

**Affiliations:** 1 Chalapathi University, A.R. Nagar, Mothadaka, Guntur 522016, Andhra Pradesh, India; 2 Department of Pharmaceutical Analysis, Chalapathi Institute of Pharmaceutical Sciences, Chalapathi Nagar, Lam, Guntur 522034, Andhra Pradesh, India; 3 Department of Pharmaceutics, Chalapathi Institute of Pharmaceutical Sciences, Chalapathi Nagar, Lam, Guntur 522034, Andhra Pradesh, India; 4 Department of Pharmacy Practice, Chalapathi Institute of Pharmaceutical Sciences, Chalapathi Nagar, Lam, Guntur 522034, Andhra Pradesh, India; mohanchandu1997@gmail.com

**Keywords:** hydrogel, organogel, nanogel, in situ gel, hydrogel-forming microneedle, stimuli-responsive polymer, sol–gel transition, Quality by Design, 3D bioprinting, controlled drug release kinetics, disease-targeted pharmacotherapy

## Abstract

Gel-based novel drug delivery systems (NDDS) occupy a mechanistically distinct niche among controlled-release platforms because they decouple three design variablesnetwork cross-link density, continuous-phase polarity, and stimulus sensitivitythat in particulate carriers (liposomes, polymeric nanoparticles) are often interdependent. This critical review synthesizes 102 primary and secondary sources published predominantly between 2010 and 2026 to interrogate, rather than merely catalog, how hydrogels, organogels, aerogels, nanogels, in situ gelling systems, and hydrogel-forming microneedles have been engineered for site-specific pharmacotherapy. Beyond a taxonomic overview, the review quantitatively contrasts formulation parameters sol–gel transition temperatures (typically 32–37 °C for poloxamer 407/188 systems), swelling ratios, mesh sizes, and reported drug-release half-lives across oncology, chronic diabetic wound care, ophthalmic and nasal-to-brain delivery, musculoskeletal (intra-articular) therapy, subunit vaccine depots, periodontal pocket therapy, and glucose-responsive insulin delivery. Particular attention is paid to the mechanistic basis of burst release, the porosity–mechanical-integrity trade-off inherent to interconnected hydrogel networks, and the divergence between preclinical rodent efficacy and the comparatively sparse controlled human trial data available for most gel platforms. The review concludes that while stimuli-responsive and 3D/4D-printed gel architectures have matured substantially as engineering constructs, clinical translation remains bottlenecked less by materials science than by inconsistent characterization standards, unresolved terminal-sterilization compatibility, and a paucity of head-to-head comparative trials against existing standard-of-care formulations.

## 1. Introduction

A gel, in the strict physicochemical sense codified since the mid-twentieth century, is a viscoelastic material in which a three-dimensional polymer network held together by covalent bonds, physical entanglements, ionic bridges, or hydrogen-bonded domains immobilizes a continuous liquid or gaseous phase without net dissolution. The pharmaceutical relevance of this definition was first demonstrated with poly(2-hydroxyethyl methacrylate) (pHEMA) networks proposed as biologically compatible implant materials [1], a discovery that pre-dates, by more than three decades, the modern concept of a ‘novel drug delivery system.’ What distinguishes contemporary gel-based NDDS from that founding work is not the underlying network chemistry which remains governed by the same Flory–Rehner swelling thermodynamics but the degree of engineering control now exerted over cross-link density, degradation kinetics, and environmental responsiveness. For broader context, vesicular carriers such as liposomes and niosomes carry their own well-documented formulation-stability considerations [2], and prolonged-release parenteral drug delivery more broadly encompasses a wider range of technologies than gel-based systems alone [3].

It is important to state at the outset, critically, that gels are not a homogeneous technology class. A poloxamer 407 thermogelling solution, a covalently cross-linked polyacrylamide intra-articular depot, and a 20 nm cross-linked chitosan nanogel share the semantic label ‘gel’ but differ by more than six orders of magnitude in characteristic length scale and operate via essentially unrelated release mechanisms (micellar disentanglement versus Fickian diffusion versus receptor-mediated cellular uptake, respectively). Reviews that treat ‘gels’ as a single monolithic platform therefore risk conflating design principles that are, in practice, not transferable across sub-classes. This review deliberately preserves those distinctions throughout, updating and substantially extending an earlier survey of the field by incorporating quantitative formulation data, comparative device-level examples (hydrogel-forming microneedles, injectable vaccine depots, intra-articular hydrogels), and an explicitly critical appraisal of translational bottlenecks.

Sources for this review were identified through iterative keyword searches (combining terms such as “hydrogel,” “organogel,” “aerogel,” “nanogel,” “in situ gel,” and “microneedle” with individual disease-domain and design-parameter terms) and were included if they were peer-reviewed, English-language, part of the primary or secondary literature addressing the engineering, formulation, or disease-specific application of a gel-based drug delivery platform, with an emphasis on publications from 2010 onward; a small number of earlier foundational sources are retained where they establish the conceptual basis of the field. Sources were excluded if they addressed gel materials exclusively in non-drug-delivery contexts (e.g., structural or purely cosmetic applications), were not peer-reviewed (conference abstracts, non-peer-reviewed preprints, and patents were excluded except where a peer-reviewed equivalent was unavailable), or were not accessible in English. This selection process was not conducted under a formal systematic-review protocol (e.g., PRISMA), consistent with this article’s stated scope as a narrative critical review rather than a systematic review; see Section 7.5 for a discussion of the limitations this entails.

Comprehensive syntheses of hydrogel-based delivery across oral, injectable, topical, and ocular routes [4], of hydrogel wound-management systems [5], and of biomedical hydrogel applications more broadly [6] and of polymer-based hydrogels in drug delivery more generally [7], collectively confirm that the field’s center of gravity has shifted from descriptive materials characterization toward disease-specific functional validation, typically in rodent models and, in a minority of cases, early-phase human trials. Section 2 re-derives a mechanistically grounded classification scheme; Section 3 traces the chronological innovation record; Section 4 dissects design strategy at the level of cross-linking chemistry and manufacturing; Section 5 critically maps ten disease domains onto specific gel architectures with quantitative examples; Section 6 catalogs characterization methodology; and Section 7 offers a candid appraisal of what remains unresolved.

## 2. Mechanistic Classification of Gel-Based Drug Delivery Systems

**Figure 1:**
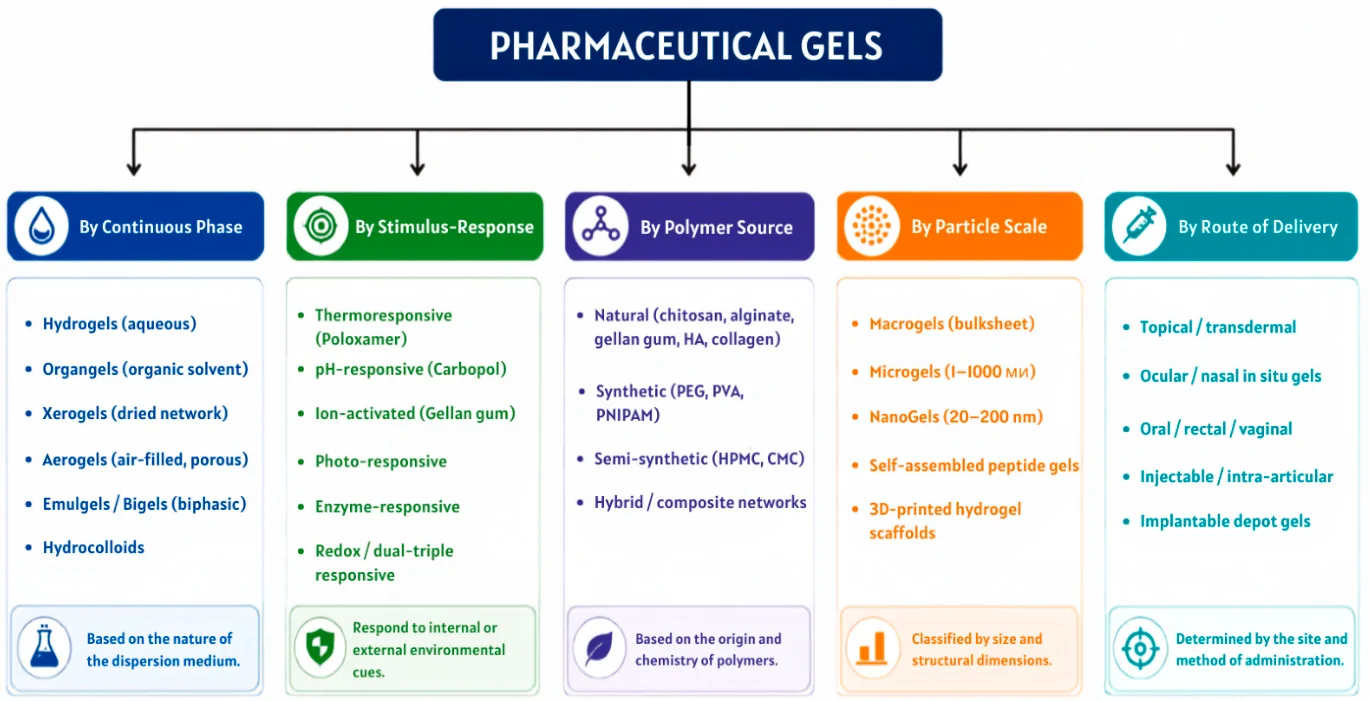
Classification of gel-based drug delivery systems in contemporary pharmaceutical practice.

A robust classification of gels must simultaneously specify (i) the chemical identity of the continuous phase, (ii) the cross-linking mechanism, and (iii) the characteristic length scale, because these three axes jointly determine the release mechanism. Comparative analyses of organogels against hydrogels emphasize that swelling-phase polarity alone reclassifies a nominally similar polymer network into a materially distinct drug carrier with different permeation, thermal-stability, and loading-capacity profiles [8], while gel-based local-anesthetic depot platforms illustrate that hydrogels, organogels, and biphasic bigels are not interchangeable but rather occupy distinct application niches dictated by drug logP and required residence time [9]. Figure 1 summarizes this multi-axis scheme.

### 2.1. Hydrogels

Hydrogels remain the dominant sub-class by publication volume. Their release behavior is governed principally by mesh size (ξ), typically 5–100 nm for chemically cross-linked networks, relative to the hydrodynamic radius of the entrapped drug; systematic control of pore interconnectivity through porogen-leaching or freeze-drying strategies has been shown to shift release from purely diffusive to a combined diffusion–erosion regime [10]. Critically, however, increasing pore interconnectivity to accelerate diffusive transport almost invariably reduces compressive modulus, a trade-off that persists across essentially every porous hydrogel system reported to date and that has not been solved by any single cross-linking chemistry [11]. Complementary reviews of hydrogel-based sustained-delivery platforms reach a broadly convergent conclusion regarding this trade-off [12].

### 2.2. Organogels

Organogels replace the aqueous continuous phase with an apolar or semi-polar organic solvent (isopropyl myristate, mineral oil, medium-chain triglycerides) immobilized by low-molecular-weight gelators or amphiphilic block copolymers such as Pluronic-lecithin combinations. Because the continuous phase is lipophilic, organogels achieve markedly higher loading capacity for logP > 3 drugs and can incorporate penetration enhancers without phase separation, a documented advantage for transdermal delivery of poorly water-soluble actives [13]. Lecithin-linked microemulsion-based organogels have additionally been engineered to extend the release half-life of hydrophilic model drugs several-fold relative to a simple microemulsion by imposing an additional gel-network diffusion barrier [14], and microemulsion-based vaginal organogels of fluconazole have demonstrated superior ex vivo mucosal retention compared with conventional cream formulations [15]. Broader technical overviews of organogel-based topical delivery similarly catalog lecithin- and sorbitan-ester-based gelator systems as the dominant formulation approach [16,17], and organogels formulated with pharmaceutical excipients such as polyvinyl alcohol and dimethyl sulfoxide further extend the achievable design space for poorly water-soluble actives [18].

### 2.3. Aerogels

Aerogels are produced by replacing the pore liquid of a hydrogel or alcogel precursor with air via supercritical CO_2_ drying, yielding densities as low as 0.05–0.3 g/cm^3^ and specific surface areas exceeding 300 m^2^/g. Polysaccharide aerogels based on alginate, chitosan, and agarose retain the biocompatibility of their hydrogel precursors while offering markedly faster initial dissolution and higher achievable drug loading per unit mass, an advantage specifically exploited for the oral and pulmonary delivery of poorly soluble actives [19]. The broader aerogel platform literature further documents that scCO_2_-processed aerogels can be tuned across three orders of magnitude in pore diameter simply by varying supercritical drying pressure and polymer concentration, though the reproducible pilot-scale manufacture of pharmaceutical-grade aerogel batches remains comparatively immature relative to hydrogel manufacture [20]. Comparative appraisals of aerogels against hydrogels corroborate that the two platforms remain functionally complementary rather than interchangeable [21], and dedicated reviews of aerogel-based antimicrobial therapy [22] and pulmonary-targeted aerogel delivery [23] further illustrate the breadth of the aerogel application space.

### 2.4. Nanogels, Emulgels, and Bigels

Nanogels (20–200 nm) merge hydrogel drug-loading versatility with nanoparticulate biodistribution advantages, discussed mechanistically in Section 4.3. Emulgels and bigels, formed by dispersing an emulsion (o/w or w/o) within a continuous gel phase, permit co-formulation of both hydrophilic and lipophilic actives in a single vehicle and have been specifically leveraged where dual-drug release kinetics differing by an order of magnitude are clinically required. A systematic review of bigel-based drug delivery corroborates that formulation performance in these biphasic systems is governed principally by the ratio and rheology of the constituent oleogel and hydrogel phases [24]. Table 1 consolidates the defining phase composition, representative polymers, characteristic length scale, and key functional attribute of each gel sub-class discussed above, to serve as a single point of reference for the sub-class comparisons developed throughout this review.

## 3. Chronological Innovation Record (2010–2026): A Critical Reading

**Figure 2:**
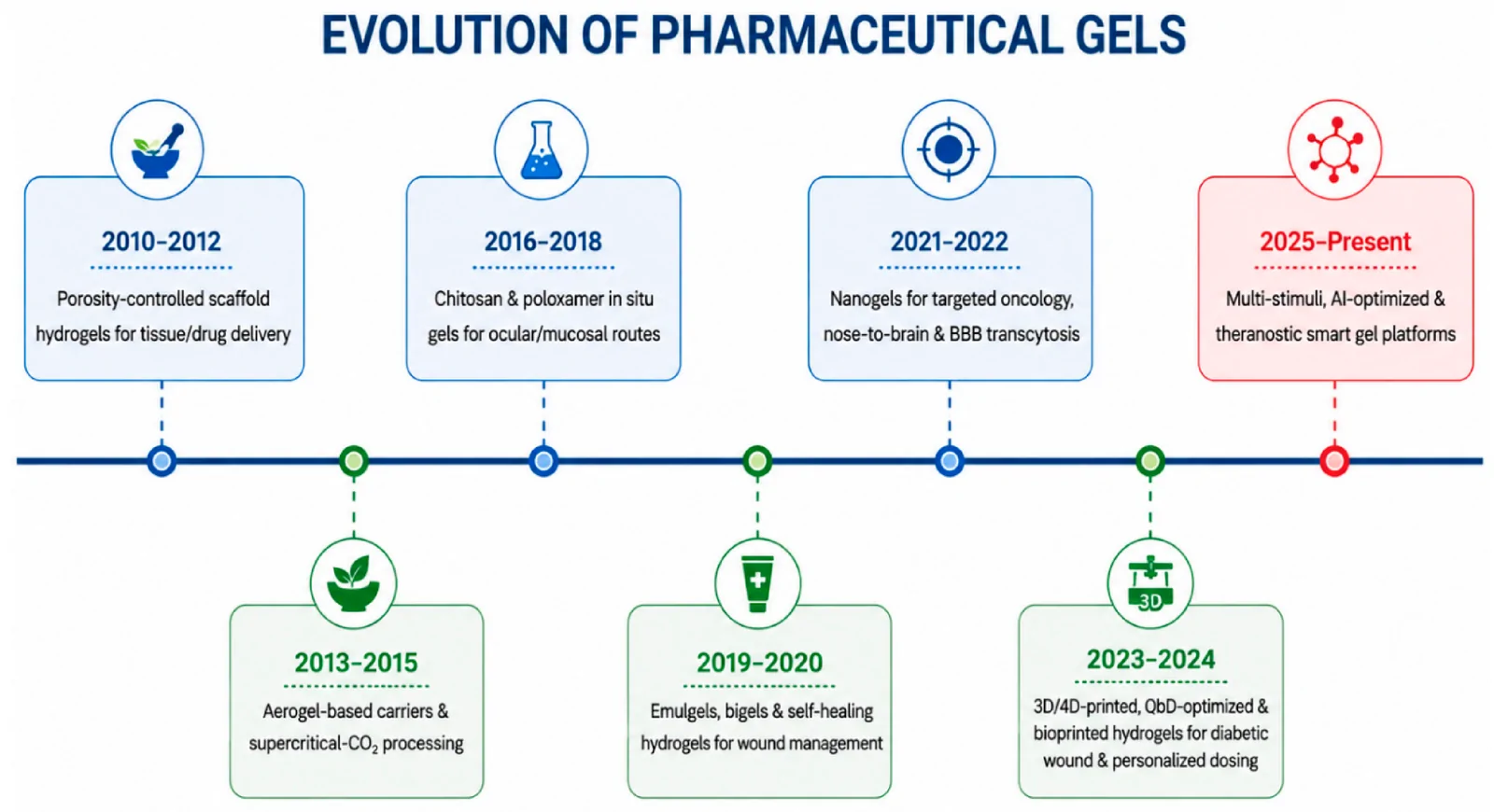
Chronological evolution of gel-based drug delivery systems (2010–present) with representative primary literature.

Framing the 2010–2026 period as a simple linear progression toward increasing sophistication would be historically inaccurate; several technology strands (hydrogel-forming microneedles, for instance) originated before 2010 [25] and were subsequently refined rather than newly invented. What genuinely changed after 2010 was the systematic coupling of these individually mature materials concepts to disease-specific functional endpoints and to digitally controlled manufacture. Early decade work concentrated on characterizing and controlling hydrogel porosity/microarchitecture as a tunable variable rather than an incidental synthesis artifact [10], alongside supercritical-CO_2_-processed aerogel carriers for oral and pulmonary delivery [19,20]. Between 2016 and 2020, poloxamer- and chitosan-based in situ gelling systems matured for ocular and nasal administration [26,27,28], and self-healing hydrogel chemistries were introduced to withstand the mechanical stresses of chronic wound dressing changes [29]. The 2021–2024 interval is best characterized as the ‘functional convergence’ phase: nanogel platforms for oncology and blood–brain barrier transcytosis matured alongside Quality-by-Design (QbD) frameworks for systematic hydrogel optimization [30,31], and 3D/4D-printed, bioprinted hydrogel scaffolds were validated specifically for diabetic wound care using patient-agnostic but wound-geometry-specific designs [32,33,34]. A bibliometric analysis of in situ gel research spanning 2003–2023 independently corroborates this trajectory, documenting a sharp rise in publication output after 2019 concentrated on formulation optimization and CNS-targeted (nose-to-brain) applications, while noting that clinical and regulatory translation has not kept pace with this formulation-stage output [35]. From 2025 onward, the literature increasingly emphasizes multi-stimuli, glucose-responsive, and vaccine-adjuvanting gel platforms [36,37,38], alongside persistent, unresolved porosity–mechanical-strength trade-offs flagged as early as the 2010–2012 period and still cited as an open problem in 2023 [11], summarized in Figure 2. Table 2 organizes these milestones chronologically by two-to-three-year periods, together with the representative primary references underpinning each innovation, to make the field’s developmental trajectory explicit rather than implicit in the narrative above.

## 4. Design and Development Strategies: A Structure–Function Analysis

**Figure 3:**
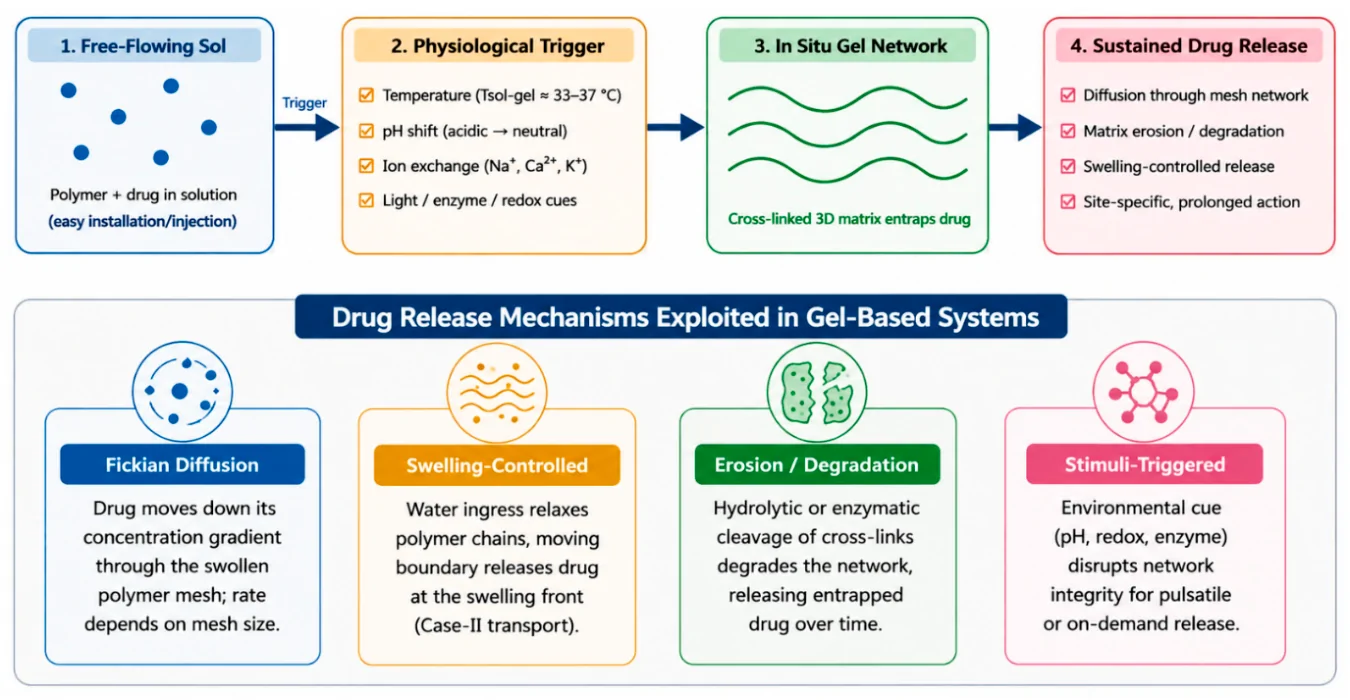
Generalized design principles and drug release mechanisms of stimuli-responsive gel-based drug delivery systems.

Figure 3 depicts the generalized design logic converting a free-flowing, administrable sol into an in vivo gel network under a physiological trigger, followed by one of four dominant release mechanisms. The sections below examine each design lever critically, with worked numerical examples drawn directly from the primary literature.

### 4.1. Polymer Selection and Cross-Linking Chemistry

Cross-linking strategy is the single largest determinant of both mechanical behavior and degradation timeline. Physical (ionic, hydrogen-bonded, hydrophobic) cross-links are reversible and shear-thinning, a property essential for injectability through fine-gauge needles, but are correspondingly more prone to premature erosion in vivo. Chemical (covalent, enzymatically triggered) cross-links confer superior mechanical robustness at the cost of injectability and, in some formulations, biodegradability [6]. Wound-management hydrogels typically favor natural polysaccharide backbones (alginate, chitosan, hyaluronic acid) precisely because their inherent bioadhesion reduces dependence on synthetic cross-linkers that could otherwise provoke local irritation [5]. Even historically simple systems illustrate the principle: a thermosensitive quaternized-chitosan/PEG hydrogel developed for nasal delivery demonstrated that varying the PEG:chitosan mass ratio alone shifted the sol–gel transition temperature by several degrees Celsius, directly altering nasal residence behavior without any change in chemical cross-link density [45]. A range of further studies illustrates the breadth of cross-linking chemistries available for hydrogel design, including rheological modeling of cross-linking behavior in polymer resins [46], UV-cross-linked poly(N-isopropylacrylamide) interpenetrated into chitosan for anti-fouling applications [47], systematic comparison of cross-linking agents for chitosan-based drug carriers [48], nonwoven-reinforced photocurable poly(glycerol sebacate) hydrogels [49], and hydrogel-conjugation chemistries engineered specifically for controlled drug delivery [50]. Injectable hydrogel design more broadly has been reviewed as a distinct formulation paradigm [51], exemplified by liposome-self-assembled injectable hydrogels for controlled release of small hydrophilic molecules [52] and by alginate hydrogels grafted with lower-critical-solution-temperature side chains engineered specifically for injectability [53].

### 4.2. Stimuli-Responsive (“Smart”) Design

Thermoresponsive poloxamer 407/188–HPMC combinations remain the most extensively validated ocular in situ gelling chemistry, exploiting a sol–gel transition typically engineered to occur between 33 °C and 35 °C below core body temperature but above ambient storage temperature so that the formulation is liquid in the vial yet gels rapidly on the ocular surface [27]. The same thermogelling principle, combined with mucoadhesive polymers such as carboxymethylcellulose and cyclodextrin-based solubility enhancers, has been extended to nasal formulations; one representative CNS-targeted formulation combining poloxamer 407/188 with PEG 6000 and hydroxypropyl-β-cyclodextrin achieved a gelation temperature of approximately 32.7 °C with a gelation time near 53 s, illustrating the fine-grained tunability achievable through ternary polymer blending [41]. Nano-silver-loaded poloxamer nasal matrices further demonstrate that incorporating inorganic antimicrobial nanoparticles does not necessarily compromise thermogelling behavior provided nanoparticle loading remains below a critical volume fraction [39]. Critically, however, nearly all thermoresponsive in situ gel data in the literature derive from in vitro or ex vivo mucosal models; sol–gel transition temperature measured in a rheometer under quiescent conditions is not necessarily equivalent to gelation behavior under the shear and dilution conditions of an actively secreting mucosal surface, a distinction that is inconsistently addressed across the reviewed formulations. Advances in stimuli-responsive injectable hydrogel design more broadly corroborate this tunability [54], and dynamic hydrogels engineered for evolving transport properties have been shown to enable self-tuning of short- and long-term cargo delivery through a related mechanism [55].

### 4.3. Nanotechnology-Integrated Gels (Nanogels)

Nanogels combine hydrogel drug-loading versatility with the biodistribution advantages of nanoparticulate carriers, including enhanced permeability and retention (EPR) at tumor sites and, when surface-functionalized with targeting ligands, receptor-mediated transcytosis across the blood–brain barrier. Diphtheria-toxin-receptor-functionalized nanogels and angiopep-2-modified nanogels have each been used to improve central nervous system penetration of chemotherapeutics, while dual- and triple-stimuli-responsive nanogel architectures are increasingly designed to require the simultaneous presence of two or three tumor-microenvironment cues (acidic pH, elevated glutathione, specific proteolytic enzyme activity) before releasing payload, a strategy intended to reduce off-target toxicity relative to single-stimulus designs [31]. The mechanistic trade-off, rarely made explicit in the primary literature, is that each additional required stimulus increases formulation complexity and batch-to-batch variability while only incrementally improving selectivity beyond what is achievable with EPR-mediated passive accumulation alone. Comprehensive reviews of nanogel-based drug delivery corroborate this design logic across a broader range of biomedical applications [56,57], a maleimide-modified hyaluronic acid nanogel-based hydrogel further demonstrates variable, tunable swelling and drug-encapsulation behavior achievable through this design class [58], and stimuli-responsive nanogels are increasingly positioned as a smart-material platform spanning biomedical applications beyond drug delivery alone [59].

### 4.4. Quality by Design (Qbd) and Additive Manufacturing

QbD frameworks formally link a predefined Quality Target Product Profile to Critical Material Attributes (polymer molecular weight, cross-linker stoichiometry) and Critical Process Parameters (mixing shear rate, curing temperature/time) through structured Design-of-Experiments, allowing formulators to define a validated design space rather than a single fixed formulation [30]. A central-composite-design-assisted QbD approach has similarly been applied to the formulation of an oral herbal gastro-retentive in situ gel, underscoring the generality of this framework beyond parenteral and topical hydrogels alone [60]. In parallel, extrusion-based 3D printing and direct-write bioprinting enable geometry-matched hydrogel scaffolds: a curcumin-loaded gelatin methacryloyl (GelMA) bioink reduced reactive-oxygen-species-induced apoptosis of adipose-derived stem cells and improved graft survival in a diabetic wound model [33], while a bioinspired 3D-printed scaffold embedding zinc-oxide nanofibrous microspheres achieved regenerative outcomes in diabetic wounds attributable jointly to antimicrobial and angiogenic mechanisms [34]. A 3D-printed egg-white hydrogel, notably, achieved proangiogenic and pro-collagen-deposition effects in diabetic wound healing using only the intrinsic bioactivity of a food-derived protein matrix, without any exogenous growth factor supplementation, a materially significant finding because it decouples therapeutic efficacy from the cost and regulatory burden of recombinant protein incorporation [32].

### 4.5. Hydrogel-Forming Microneedles: A Convergent Device–Gel Technology

Hydrogel-forming microneedle arrays, first systematically described using cross-linked poly(methyl vinyl ether-co-maleic acid) matrices, penetrate the stratum corneum as dry, mechanically rigid structures that subsequently imbibe interstitial fluid and swell in situ, opening transient aqueous conduits through which a co-formulated drug reservoir can diffuse [25]. This two-phase mechanical/hydrogel behavior is functionally distinct from either solid or dissolving microneedle designs and specifically addresses the mechanical-fragility limitation that otherwise restricts hydrogel use to non-penetrating routes. Applied to insulin delivery, hydrogel and glucose-responsive microneedle patches have been engineered around glucose-oxidase-mediated local pH shifts; one self-crosslinkable matrix microneedle patch demonstrated a polymer pKa of 6.86 tuned close to physiological pH specifically to maximize glucose sensitivity at clinically relevant concentrations [36], while a layer-by-layer pH-responsive insulin/poly-L-glutamic-acid coating achieved insulin release within one minute of skin insertion, with pharmacokinetic and pharmacodynamic profiles comparable to subcutaneous injection [37]. A comprehensive review of microneedle-based insulin delivery nonetheless concludes that scale-up manufacturing consistency and long-term glucose-responsive stability remain unresolved translational barriers common to hollow, dissolving, and hydrogel-forming microneedle designs alike [61]. A dedicated review of hydrogel-based microneedle delivery systems more broadly corroborates this translational assessment across disease indications beyond insulin delivery alone [62].

## 5. Disease-Targeted Therapeutic Applications: A Critical, Example-Driven Survey

Table 3 summarizes ten disease domains in which gel-based platforms have demonstrated at least preclinical proof of concept; each is discussed with specific quantitative examples below.

### 5.1. Oncology

Polysaccharide-based hydrogels loaded with chemotherapeutics improve tumor-localized exposure while reducing systemic toxicity relative to bolus intravenous administration [63]. Nanogels functionalized with targeting ligands enable receptor-mediated transcytosis across the blood–brain barrier for glioma therapy and permit co-delivery of chemo-immunotherapeutic combinations, such as paclitaxel with interleukin-2, for solid tumors including triple-negative breast cancer [31]. At the device-materials interface, an injectable hydrogel embedding cowpea mosaic virus nanoparticles has been shown to arrest colon cancer growth in a murine model through a combined immunostimulatory and localized-retention mechanism, illustrating that plant-virus nanoparticle immunogenicity can be repurposed within a gel depot as an anticancer, rather than purely vaccine, modality [65]. A dedicated Special Issue on gel-based cancer drug delivery corroborates that topical hydrogel formulations for skin cancer and transliposomal nanocarrier gels for localized cytotoxic delivery represent an active, clinically motivated sub-field. Injectable hydrogels have separately been reviewed as an emerging platform specifically for tumor-directed drug delivery [66], and biopolymer-based aerogels are similarly being explored as smart, targeted platforms for cancer treatment [67], a therapeutic breadth echoed by a companion editorial on the same Special Issue [64].

### 5.2. Diabetic Chronic Wound Healing

Diabetic wounds present a self-perpetuating pathology of impaired angiogenesis, elevated reactive oxygen species, and chronic low-grade infection that conventional gauze dressings cannot interrupt. Further, 3D-printed bioactive hydrogels, including a food-protein-derived egg-white matrix requiring no exogenous growth factor, have demonstrated proangiogenic effects sufficient to accelerate healing in murine diabetic wound models [32]. A curcumin-loaded GelMA bioink reduced ROS-induced apoptosis of transplanted adipose-derived stem cells specifically by scavenging free radicals within the printed scaffold microenvironment [33], and a bioinspired black-phosphorus hydrogel combined antibacterial photothermal activity with antioxidant capacity in a single stepwise-acting matrix [68]. Complementary approaches include mussel-inspired adhesive, antibacterial, thermosensitive hydroxybutyl chitosan hydrogels used as three-dimensional culture matrices for mesenchymal stem cells during wound regeneration [91], personalized 3D scaffolds with controlled fiber alignment for mesenchymal-stem-cell-laden diabetic wound treatment [92], injectable self-assembling peptide nanofiber hydrogels that provide a bioactive three-dimensional platform for chronic wound tissue regeneration without requiring external cross-linking agents [93], and dual-cross-linked hydrogel patches engineered specifically to withstand the mechanical shear of diabetic foot dressing changes while sustaining drug release [94]. Critically, essentially all of these outcomes derive from rodent full-thickness excisional wound models; translation to the substantially larger, more vascularly compromised, and microbiologically polymicrobial human diabetic foot ulcer has not yet been demonstrated with comparable rigor across most of these systems. Additional reported examples include a three-dimensional living dressing shown to improve healing and modulate the immune response in a thermal-injury model [95], silver-nanoparticle-doped PVA hydrogels loaded with ibuprofen for combined antibacterial and analgesic action [69], a ciprofloxacin-loaded hydrogel scaffold engineered for sustained antibiotic release [70], a rapidly forming double-cross-linked network hydrogel developed specifically to promote wound healing [71], and a piezoelectric, conductive, injectable hydrogel that promotes wound healing through electrical stimulation [72]. Two further reviews specifically synthesize the multifunctional-hydrogel literature for diabetic wound healing [73] and the use of multifunctional nanocomposite-mediated hydrogels for diabetic wound repair [74].

### 5.3. Ophthalmic Delivery

Conventional eye drops achieve bioavailability below approximately 5% owing to rapid nasolacrimal drainage and the blink reflex. Gel-based ophthalmic materials, particularly thermosensitive poloxamer/HPMC in situ gels, substantially prolong ocular surface residence and enable sustained release of both small-molecule and macromolecular drugs [26,27], and chitosan-based in situ gels additionally exploit inherent mucoadhesive and corneal-wound-healing bioactivity, a dual pharmacological/formulation function distinct from an inert poloxamer matrix [28]. Dedicated reviews of in situ gelling systems for ocular drug delivery [96] and of niosomal in situ gels for ocular applications specifically [97] further corroborate the breadth of formulation strategies available within this application area.

### 5.4. Nasal and CNS-Targeted Delivery

In situ nasal gels combine spray-based ease of administration with prolonged mucosal residence; incorporation of mucoadhesive polymers, cyclodextrin solubilizers, and nanocarriers within thermogelling poloxamer matrices increasingly enables nose-to-brain transport for CNS-acting drugs that would otherwise be excluded by the blood–brain barrier [41]. Nano-silver-loaded poloxamer nasal matrices exemplify how antimicrobial and thermogelling functions can be integrated within a single nasal platform without loss of gelation fidelity [42].

### 5.5. Dermatological and Topical Applications

Organogels and lecithin/proniosomal gel systems enhance transdermal permeation of both hydrophilic and lipophilic actives by combining a penetration-enhancing continuous phase with gel-network sustained release. Proniosome-based transdermal delivery of piroxicam demonstrated markedly improved skin flux relative to a conventional gel base, attributable to the surfactant-vesicle intermediate formed on skin hydration [75], and lecithin-linked microemulsion gelatin gels extended hydrophilic model-drug release several-fold by superimposing a gel-diffusion barrier atop the microemulsion droplet reservoir [14]. A broader review of hydrogel-based transdermal delivery corroborates the general advantages of gel-network sustained release at the skin surface [76], and disease-specific lecithin-organogel applications including topical treatment of psoriasis [77], ionic-liquid-mediated transdermal delivery of cyclosporine A for psoriasis [78], and lecithin organogels more broadly as a carrier for skin disease treatment [79] further illustrate the clinical breadth of this platform.

### 5.6. Gastrointestinal, Rectal, and Vaginal Mucosal Therapy

Mucoadhesive and pH-responsive polymer gels extend residence time at GI, rectal, and vaginal mucosal surfaces. A microemulsion-based vaginal gel of fluconazole demonstrated superior in vitro release and ex vivo mucosal retention relative to a conventional vaginal cream, attributed to the combined solubilizing and mucoadhesive action of the microemulsion–gel hybrid [15], while sprayable hydrogel sealants applied directly to gastrointestinal wound surfaces provide an emerging, minimally invasive alternative to surgical closure for shielding compromised mucosa during healing [80]. Rapid in situ forming PEG hydrogels have similarly been engineered specifically for mucosal drug delivery [81], and a broader review of targeted hydrogel delivery through the human gastrointestinal tract corroborates the mucosal-retention advantages described above [82].

### 5.7. Musculoskeletal Therapy: Intra-Articular Osteoarthritis Gels

Osteoarthritis (OA) pharmacotherapy is fundamentally limited by rapid synovial clearance of intra-articularly injected drugs, necessitating frequent re-injection. Injectable hydrogels engineered specifically to extend intra-articular residence, including thermosensitive chitosan hydrogels providing lubrication and reducing cartilage-surface friction, and gene-vector-loaded alginate hydrogels delivering insulin-like growth factor-1-encoding adeno-associated virus for cartilage repair, have each demonstrated significant reduction in inflammatory cytokine expression (TNF-α, IL-1β, IL-6, IL-17) and improved cartilage-repair histology relative to hyaluronic acid viscosupplementation alone in animal OA models [39]. This musculoskeletal application is mechanistically distinct from the wound-healing and ophthalmic examples above in that the hydrogel here must simultaneously act as a load-bearing, self-healing lubricant and as a drug/gene/cell reservoir a dual mechanical–pharmacological requirement rarely encountered elsewhere in gel-based NDDS design. Broader reviews of intra-articular injectable biomaterials for cartilage repair and regeneration [83] and of responsive hydrogel-based platforms specifically engineered for osteoarthritis treatment [84] corroborate this musculoskeletal application space.

### 5.8. Vaccination and Immunotherapy

Injectable hydrogel depots address a structural limitation of conventional bolus subunit vaccination: rapid antigen clearance limits the duration of germinal-center reactions required for high-affinity antibody maturation. A high-affinity injectable hydrogel scaffold engineered for sustained delivery of a SARS-CoV-2 receptor-binding-domain (RBD) subunit vaccine achieved serum IgG titers comparable to three separate bolus injections from a single administration, while additionally polarizing the response toward a TH1-associated IgG2b profile and sustaining robust germinal-center reactions [38]. This finding is mechanistically significant because it demonstrates that a purely physical formulation intervention prolonging local antigen residence can substitute, at least in a murine model, for repeat-dose immunization schedules, with direct implications for vaccine logistics in resource-limited settings. Separately, an injectable hydrogel embedding plant-virus nanoparticles has demonstrated dual utility as both a vaccine-adjuvant platform and, as noted in Section 5.1, an anticancer immunostimulatory depot [65]. Broader reviews of hydrogel-based immunotherapy delivery [85] and of immunomodulatory hydrogel-guided vaccine delivery systems [86] corroborate this convergence, and a regimen-compression strategy leveraging an injectable hydrogel depot has separately been shown to sustain commercial-vaccine antigen exposure with fewer administrations [40].

### 5.9. Periodontal Disease

Periodontal pockets present a uniquely challenging local-delivery environment owing to continuous gingival crevicular fluid flow, which rapidly clears conventional topical antimicrobial formulations. In situ gelling systems that transition from a low-viscosity sol to a gel upon contact with pocket fluid, triggered by temperature, pH, or ionic strength, substantially improve intra-pocket antimicrobial residence relative to irrigation alone [87]. A comprehensive narrative review of periodontal in situ gel therapy further catalogs gellan gum, alginic acid, xyloglucan, and chitosan as the dominant polymer choices, but explicitly flags inconsistent gelation reproducibility, low mechanical strength once formed, and a scarcity of long-term controlled clinical trial data as persistent barriers to routine clinical adoption [43], an appraisal consistent with the broader translational critique developed in Section 7. A further review of periodontal-pocket pathogenesis, treatment, and available in vitro/in vivo models corroborates these persistent translational barriers [44].

### 5.10. Endocrine Disease: Glucose-Responsive Insulin Delivery

Basal-bolus insulin therapy via subcutaneous injection remains burdened by injection-associated non-adherence. Hydrogel-forming and hydrogel-based glucose-responsive microneedle patches address this by coupling mechanical skin penetration to a chemically responsive release trigger, discussed mechanistically in Section 4.5 [25,36,37,61]. The clinical significance of this convergence of device technology (microneedles) merged with stimuli-responsive gel chemistry is that it simultaneously solves two independent translational problems (needle-associated pain and imprecise dosing) that neither technology solves alone. Glucose-responsive concanavalin-A-based smart hydrogels offer a related, chemically distinct route to controlled insulin delivery [88], a broader systematic review corroborates the range of glucose-responsive drug delivery systems developed for diabetes management [89], and glucose-responsive microneedle-based systems specifically engineered for transdermal insulin delivery further illustrate the convergence of these design principles [90].

## 6. Characterization Methodology: Standards and Inconsistencies

Robust physicochemical and biological characterization underpins both formulation optimization and regulatory acceptance of gel-based NDDS. QbD-driven development explicitly links Critical Material Attributes and Critical Process Parameters to defined characterization outputs [30], and comprehensive hydrogel reviews similarly emphasize systematic evaluation spanning synthesis, swelling, and in vivo performance [4]. Table 4 lists key characterization methods; Table 5 then presents representative quantitative outcomes reported for specific formulations discussed in Section 5, illustrating the substantial variability in reported gelation temperature, release duration, and drug loading even within a single gel sub-class.

Beyond cataloging methods, the data in Table 5 expose a deeper problem: characterization outputs are rarely comparable across studies because the underlying protocols are not standardized. Gelation temperature and time are reported using divergent methods, oscillatory rheometry versus tube-inversion or vial-tilt tests with different strain rates, ramp rates, and endpoint criteria, so values are not strictly comparable across formulations [41]. Drug-release characterization shows a parallel gap: Franz diffusion cells, dialysis-bag methods, and USP dissolution apparatus differ in sink conditions, membrane permeability, and hydrodynamics, so release-kinetic fits (zero-order, Higuchi, Korsmeyer–Peppas) drawn from different apparatus are not directly transferable between formulations [51]. Network structure and mechanical data are similarly method-dependent: SEM/TEM pore-size measurements vary with sample preparation (freeze-drying versus critical-point drying), and mechanical testing lacks agreed strain rates or probe geometries across the reviewed literature, making reported elasticity and adhesiveness values study-specific rather than field-wide benchmarks [4]. This matters for translation: QbD frameworks tie Critical Material Attributes to defined characterization outputs [30], but that link only holds if characterization itself is standardized. A harmonized minimum reporting protocol specifying instrument settings, sample preparation, and endpoint definitions for rheology, drug release, and mechanical testing would let the variability in Table 5 reflect genuine formulation differences rather than methodological artifacts.

## 7. Critical Appraisal: Translational Bottlenecks and Unresolved Questions

Four recurring, mechanistically linked problems emerge on critical reading of the literature synthesized above, and none is fully solved by any single gel sub-class or manufacturing strategy.

### 7.1. The Porosity–Mechanical-Integrity Trade-Off Is Structural, Not Incidental

Increasing hydrogel pore interconnectivity accelerates diffusive drug and cell transport but reduces compressive and tensile modulus, an inverse relationship documented across essentially every porous hydrogel chemistry examined to date and still explicitly identified as unresolved in materials reviews published as recently as 2023 [11]. No cross-linking strategy reviewed in this article physical, covalent, or enzymatic escapes this trade-off; formulations instead select a point on the trade-off curve appropriate to their specific mechanical-loading requirement (e.g., intra-articular hydrogels prioritize mechanical integrity under joint loading [39], whereas ocular in situ gels prioritize diffusive release over mechanical strength).

### 7.2. In Vitro Gelation Behavior Does Not Reliably Predict In Vivo Performance

Sol–gel transition temperatures and gelation times reported under quiescent rheometric conditions [41] are measured in the absence of the continuous mucosal secretion, dilution, and mechanical shear present at the intended application site. This discrepancy is rarely quantified directly in the primary literature, representing a methodological gap that likely contributes to the frequently observed divergence between promising in vitro release profiles and more modest in vivo residence times.

### 7.3. Preclinical Rodent Efficacy Substantially Outpaces Human Clinical Validation

Across oncology [31,65], diabetic wound care [32,33,34,68], vaccination [38], and periodontal therapy [43,87], the large majority of quantitative efficacy data derive from murine or, less commonly, rabbit and porcine models. Rodent skin, synovial volume, and immune architecture differ substantially from human equivalents in ways directly relevant to gel performance (e.g., murine skin lacks the stratum corneum thickness of human skin, and murine joint volumes are orders of magnitude smaller than human knee synovial volume), such that reported efficacy magnitudes should be interpreted as proof-of-mechanism rather than as predictive of human dosing or duration of effect.

### 7.4. Characterization Standardization Remains Incomplete Across the Field

As Table 5 illustrates, formulations are characterized using non-uniform combinations of gelation, mechanical, and release metrics, making direct cross-study comparison difficult even within a single application area. A dedicated periodontal in situ gel review explicitly identifies inconsistent gelation reproducibility and a scarcity of long-term controlled clinical data as barriers specific to that application [43], and the broader in situ gel bibliometric analysis confirms that formulation-stage publication output has grown far faster than clinical-translation output across the field as a whole [35]. QbD frameworks partially address this by formalizing Critical Quality Attributes [30], but QbD adoption itself remains inconsistent across the academic literature reviewed here, being considerably more common in industry-sponsored than in purely academic formulation studies. A broader synthesis of the translational and biomedical application literature reaches a consistent conclusion regarding this standardization gap [98].

Looking forward, four converging trends are likely to shape the next generation of gel-based drug delivery: (i) multi-stimuli nanogels capable of discriminating between overlapping pathological cues to improve selectivity beyond passive EPR accumulation [31]; (ii) digitally controlled 3D/4D bioprinting enabling patient- and wound-geometry-specific constructs with embedded sensing capability [32,33]; (iii) machine-learning-assisted Design-of-Experiments layered atop existing QbD frameworks to accelerate formulation screening [30]; and (iv) convergent device–gel platforms, exemplified by hydrogel-forming and glucose-responsive microneedles, that combine mechanical and chemical functionality within a single construct [25,36,37,61]. A further, related trend is the growing availability of commercial hydrogel products spanning multiple administration routes, underscoring that industrial translation is already underway for at least a subset of gel platforms [99,100], alongside an early but expanding literature applying hydrogel engineering principles to flexible bioelectronic and sensing applications beyond conventional drug delivery [101,102]. None of these trends, however, addresses the standardization and human-translation gaps identified above, which are methodological and regulatory rather than purely materials-scientific in origin.

### 7.5. Limitations of This Review

This review has methodological limitations distinct from the translational bottlenecks of the field itself, discussed above. First, source identification relied on keyword-based searching rather than a formal systematic-review protocol (e.g., PRISMA), and, as with the great majority of narrative reviews in this space, was restricted to English-language, peer-reviewed publications; relevant work published in other languages, and findings disclosed only in the patent literature, industry white papers, or regulatory filings, are therefore not captured here. Second, the reviewed literature is itself subject to publication bias toward positive efficacy outcomes, meaning that formulations with disappointing or null results in vivo are systematically under-represented relative to their true prevalence in the experimental record. Third, this review synthesizes reported findings rather than independently re-analyzing primary data, so apparent cross-study consistency in a given formulation parameter may in some cases reflect shared methodological convention (e.g., a common assay protocol) rather than a true underlying material property. Readers should weigh the conclusions drawn in Section 7 accordingly.

## 8. Conclusions

Gel-based drug delivery systems have matured from a single conceptual material, the hydrophilic pHEMA network of the 1960s, into a mechanistically diverse family spanning hydrogels, organogels, aerogels, nanogels, in situ gelling systems, and hydrogel-forming microneedles. A broader synthesis of achievements and future directions in hydrogel-based therapeutics corroborates this trajectory of engineering maturation. Rather than restating the barriers cataloged in Section 7, the priority now is execution: the field does not principally need new network chemistries so much as it needs the standardized characterization protocols, larger-animal and early-phase human comparative trials, and wider QbD adoption identified there to be applied consistently across sub-classes and research groups. On that basis, three developments are most likely to determine the pace of clinical translation over the next several years: consolidation around a small number of validated characterization standards per gel sub-class, so that formulations become genuinely comparable across studies; a shift from single-arm preclinical efficacy reporting toward head-to-head trials against existing standard-of-care formulations; and closer integration of digitally controlled manufacture (3D/4D printing, QbD-guided Design-of-Experiments) with regulatory-grade sterilization and stability testing from the earliest stages of formulation development, rather than as a late-stage afterthought. Gel-based platforms are, on the evidence synthesized here, engineering-mature; what remains is chiefly an evidentiary and standardization gap rather than a materials-science one.

## Figures and Tables

**Table 1:** Representative gel types, defining features, and typical pharmaceutical polymers.

Gel Type	Continuous/Dispersed Phase	Representative Polymers/Gelators	Characteristic Length Scale	Key Functional Attribute
Hydrogel	Water	PVA, PEG, HPMC, chitosan, alginate, gellan gum	Bulk (mm–cm); mesh 5–100 nm	High water content; tunable swelling; biocompatible
Organogel	Organic solvent/oil	Lecithin, sorbitan esters, Pluronic-lecithin (PLO)	Bulk; micellar domains 5–50 nm	High lipophilic drug loading; enhanced skin permeation
Aerogel	Air (nanoporous solid)	Alginate, chitosan, agarose, silica	Pores 2–50 nm; SSA >300 m^2^/g	Ultra-low density; very high specific surface area
Xerogel	Dried, collapsed network	Silica, cellulose derivatives	Bulk, dense	Rigid matrix; long-term implantable release
Nanogel	Water (nano-cross-linked)	Chitosan, hyaluronic acid, PNIPAM copolymers	20–200 nm	Tumor/BBB targeting; EPR-mediated accumulation
In situ gel	Sol at RT → gel in vivo	Poloxamer 407/188, gellan gum, chitosan	Bulk, mucosal-conforming	Ease of instillation; prolonged mucosal residence
Emulgel/Bigel	Biphasic (o/w or w/o + gel)	Carbopol + lipid-phase blends	Droplets 1–20 µm in gel matrix	Dual hydrophilic/lipophilic co-loading
Hydrogel-forming microneedle	Cross-linked polymer, dry-swelling	PMVE/MA, HA, PVA composites	Needle 150–900 µm	Painless transdermal macromolecule delivery

**Table 2:** Milestone innovations in gel-based drug delivery, 2010 to present, with representative primary references.

Period	Milestone Innovation	Representative Reference(s)
2010–2012	Systematic control of hydrogel porosity/microarchitecture for tunable release	[10]
2013–2015	Supercritical-CO_2_-processed polysaccharide aerogel carriers	[19,20]
2016–2018	Maturation of poloxamer/chitosan in situ gels for ocular and mucosal delivery	[26,27,28]
2019–2020	Self-healing hydrogels and glucose-responsive smart microneedle patches	[29,37]
2021–2022	Nanogels for targeted oncology/BBB transcytosis; intra-articular OA hydrogels	[31,39]
2023–2024	QbD-optimized, 3D/4D-printed bioprinted hydrogels; injectable vaccine depots	[30,32,38,40]
2025–Present	Multi-stimuli, bibliometrically validated, periodontal and theranostic smart gels	[35,41,42,43,44]

**Table 3:** Disease-targeted gel-based drug delivery systems and their functional rationale.

Disease Area	Gel Platform	Functional Rationale	Ref(s)
Oncology (solid tumors)	Nanogels; polysaccharide hydrogels; injectable virus-nanoparticle hydrogels	EPR-mediated accumulation; BBB transcytosis; localized cytotoxic depot	[31,63,64,65,66,67]
Diabetic chronic wounds	3D-printed, self-healing and antimicrobial hydrogels	Angiogenesis promotion, ROS scavenging, sustained antimicrobial release	[29,32,33,34,68,69,70,71,72,73,74]
Ophthalmic disorders	Poloxamer/HPMC and chitosan thermosensitive in situ gels	Extended precorneal residence over conventional eye drops	[26,27,28]
CNS disorders (nose-to-brain)	Mucoadhesive thermosensitive nasal in situ gels	Olfactory/trigeminal bypass of BBB; sustained nasal residence	[41,42]
Dermatological conditions	Organogels; transdermal proniosomal/lecithin gels	Enhanced skin permeation of lipophilic actives	[8,13,14,75,76,77,78,79]
GI, rectal and vaginal mucosa	Mucoadhesive and pH-responsive hydrogels; sprayable sealants	Prolonged mucosal contact; localized sustained action	[15,80,81,82]
Musculoskeletal (osteoarthritis)	Injectable intra-articular hydrogels and hydrogel microspheres	Sustained intra-articular drug/cell/gene retention	[39,83,84]
Vaccination/immunotherapy	Injectable polymer-nanoparticle and scaffold hydrogels	Sustained antigen/adjuvant co-presentation; germinal-center priming	[38,40,65,85,86]
Periodontal disease	In situ gelling intra-pocket formulations	Localized, sustained antimicrobial release in periodontal pocket	[43,44,87]
Endocrine (diabetes/insulin)	Glucose-responsive hydrogel-forming microneedles	On-demand, minimally invasive insulin release	[25,36,37,61,88,89,90]

**Table 4:** Key characterization techniques for gel-based drug delivery systems.

Category	Technique(s)	Parameter Assessed
Rheology and gelation	Oscillatory rheometry, tube-inversion, vial-tilt method	Sol–gel transition temperature/pH, viscosity, gel strength
Network structure	SEM, TEM, AFM, mercury/N2 porosimetry	Pore size, morphology, network interconnectivity
Swelling and degradation	Gravimetric swelling ratio, mass-loss/erosion studies	Water uptake capacity, biodegradation kinetics
Mechanical properties	Texture analysis, compression/tensile testing	Elasticity, hardness, adhesiveness, spreadability
Drug release	Franz diffusion cell, dialysis bag, USP dissolution apparatus	Release kinetics (zero-order, Higuchi, Korsmeyer–Peppas)
Mucoadhesion	Texture-analyzer detachment force, ex vivo mucosal residence	Bioadhesive strength and retention time
Biocompatibility and safety	MTT/cytotoxicity assays, histopathology, sterility testing	Cell viability, tissue irritation, microbial safety
Stability	ICH-guideline accelerated and long-term stability studies	Physicochemical stability over shelf life

**Table 5:** Representative quantitative formulation data reported for specific gel systems discussed in this review.

Formulation	Reported Quantitative Outcome	Ref.
Poloxamer/PEG6000/HP-β-CD nasal in situ gel	Tgel ≈ 32.7 °C; gelation time ≈ 53 s	[41]
Self-crosslinkable glucose-responsive MN polymer	Polymer pKa ≈ 6.86 ± 0.38 (feed ratio 1:2 *w*/*w*)	[36]
Insulin/poly-L-glutamic-acid LbL-coated microneedle	Full coating dissociation in <1 min post-insertion	[37]
Injectable corticosteroid nanoparticle–hydrogel (OA)	Sustained anti-inflammatory effect over 6 weeks in vitro	[39] context
Injectable RBD subunit-vaccine hydrogel scaffold	Single dose ≈ three-bolus-injection IgG titer equivalence	[38]
Nano-silver poloxamer nasal matrix	Thermogelling retained despite nanoparticle incorporation	[42]

## Data Availability

No new data were generated or analyzed in this study. As a narrative/critical review article, this manuscript synthesizes and discusses data already reported in the primary and secondary sources cited in the Reference list; the data presented and discussed in this study are therefore available within the article and its cited references. Further clarification is available on request from the corresponding author.
